# Supplementary material for: CryoEM and stability analysis of virus-like particles of potyvirus and ipomovirus infecting a common host
Source: Commun Biol. 2023 Apr 19;6:433. doi: 10.1038/s42003-023-04799-x (PMC10115852; doi:10.1038/s42003-023-04799-x)
Supplement: Supplementary file 2 — Supplementary Material [file 42003_2023_4799_MOESM2_ESM.pdf]

## Supplementary material for:

# CryoEM and stability analysis of virus-like particles of potyvirus and ipomovirus infecting a common host

Ornela Chase[1], Abid Javed[2], Matthew J. Byrne[2]†, Eva C. Thuenemann[3], George P. Lomonossoff[3], Neil A. Ranson[2] and Juan José López-Moya[1, *]

[1]Centre for Research in Agricultural Genomics (CRAG, CSIC-IRTA-UAB-UB), 08193 Cerdanyola del Vallès, Barcelona, Spain.
[2]Astbury Centre for Structural Molecular Biology, School of Molecular and Cellular Biology, Faculty of Biological Sciences, University of Leeds, Leeds, UK.
[3] Department of Biochemistry and Metabolism, John Innes Centre, Norwich Research Park, Norwich NR4 7UH, UK.
† Current address: Electron Bio-Imaging Centre, Diamond Light Source, Harwell Science and Innovation Campus, Fermi Ave, Didcot, Oxfordshire OX11 0DE, UK.
* **Correspondence:** Juan José López-Moya (juanjose.lopez@cragenomica.es)

Contains:

- 8 Supplementary Figures (1 to 8)
- 4 Supplementary Tables (1 to 4)

**Note:** Full wwPDB EM validation Reports produced by the wwPDB biocuration pipeline after annotation of the structures corresponding to SPFMV VLP (PDB ID: 8ACB and EMDB ID: EMD-15345, deposited on 2022-07-5 with Resolution 2.60 Å) and to SPMMV VLP (PDB ID: 8ACC and EMDB ID: EMD-15346 deposited on 2022-07-5 with Resolution 2.90 Å) are available under request.

**Supplementary Fig. 1: Cross reactivities of SPFMV-CP and SPMMV-CP with homologous and heterologous CPs.**

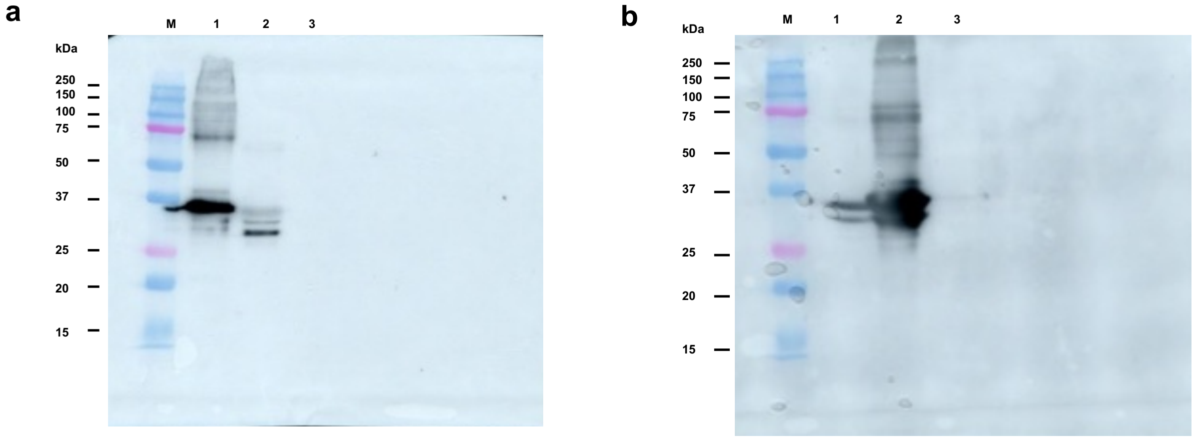


c

```
SPFMV    SSESTEFKNAGADPPAPKPKNIPPPPTITEITDPE---DPKQAALKAARAKQPAVIPESYGRDTSKERES 67
SPMMV    ---------------------ASTSKTIEELQQEMEDLDADTTITVVQRETQKARIREQIET--L-RAQQ 46

         IVGASSKGVKDKDVNV-----------GTVCTFVVPRVKMNANKKRQPMVNGRAIINFQHLSTYEPEQFE 126
         MVRP-SEAQLQPDVTPTQIVTFEPPRVTGFCALWIPRQQRNY----------MTTAYIEKIKAYVPHSNL 105

         VANTRSTQEQFQAWYEGVKGDYGV-DDTGMGILLNGLMVWCIENGTSPNING--VW-----TMMDGDEQV 188
         IESGLASEAQLTSWIENTCRDYQVSMDVFMTTVLPAWIVNCIINGTSQERTNEHTWRAVIMANMEDQEVL 175

         TYPIKPLLDHAVPTFRQIMTHFSDVAEA-YIEMRNRTKAYMPRYGLQRNLTDMSLARYAFDFYELHSTTP 257
         YYPIKPIIVNAQPTLRQVMRHFGEQAVAQYMNSLQAGKPFTVKGAVTAGYANVQDAWLGIDFLRDTMQLT 245

         ARAKEAHLQMKAAALKNARNRLFGLDGNVSTQEEDTERHTTTDVTRNIHNLLGMRGVQ 315
         TKQMEVKHQIIAANVTRRKIRVFALAAPGDGDELDTERHVVDDVARGRHSLRGAQLD- 302
```

Western blot analysis of samples corresponding to SPFMV-VLPs (lanes 1), SPMMV-VLPs (lanes 2) and the empty vector (lanes 3) using commercial polyclonal antibodies against SFPMV-CP (**a**) or against SPMMV-CP (**b**), respectively. **c** Clustal Omega amino acid alignment among SPFMV-CP and SPMMV-CP, showing 29% identity (59/206).

**Supplementary Fig. 2: Length range of SPFMV and SPMMV VLPs.**

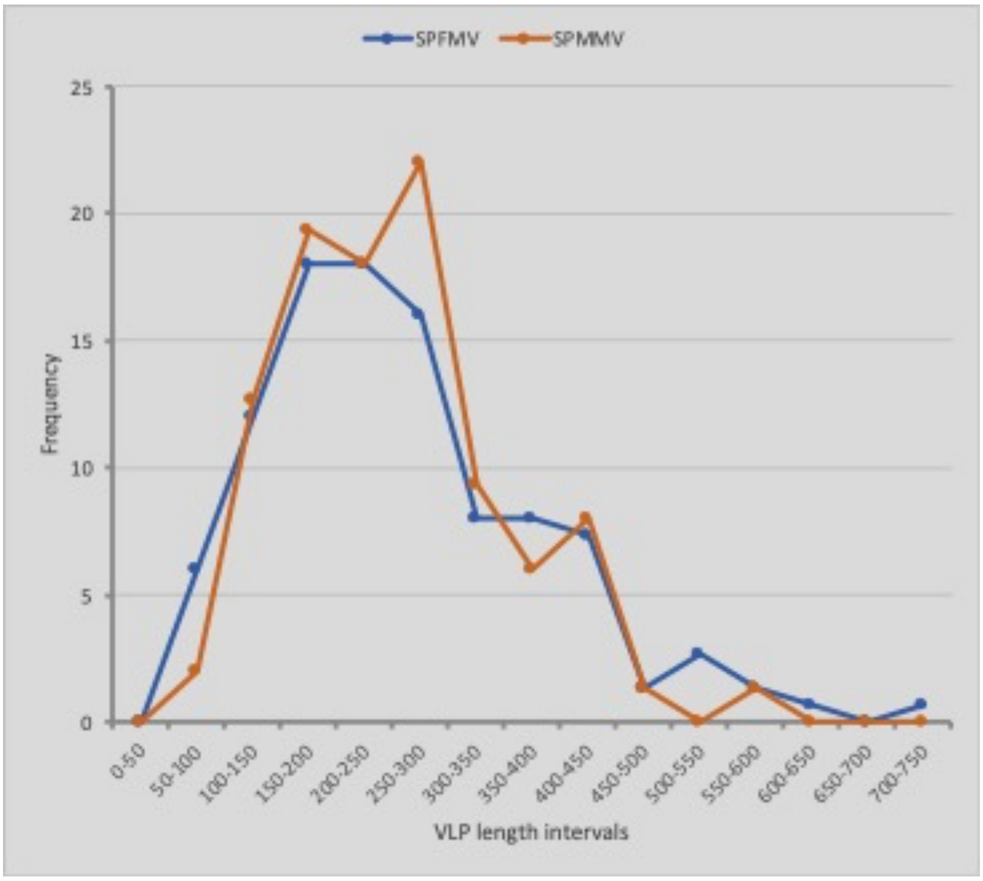


The frequency of VLPs in the corresponding intervals of length range are shown for SPFMV (blue line) and for SPMMV (orange line).

**Supplementary Fig. 3: Western blot analysis of SPMMV VLPs.**

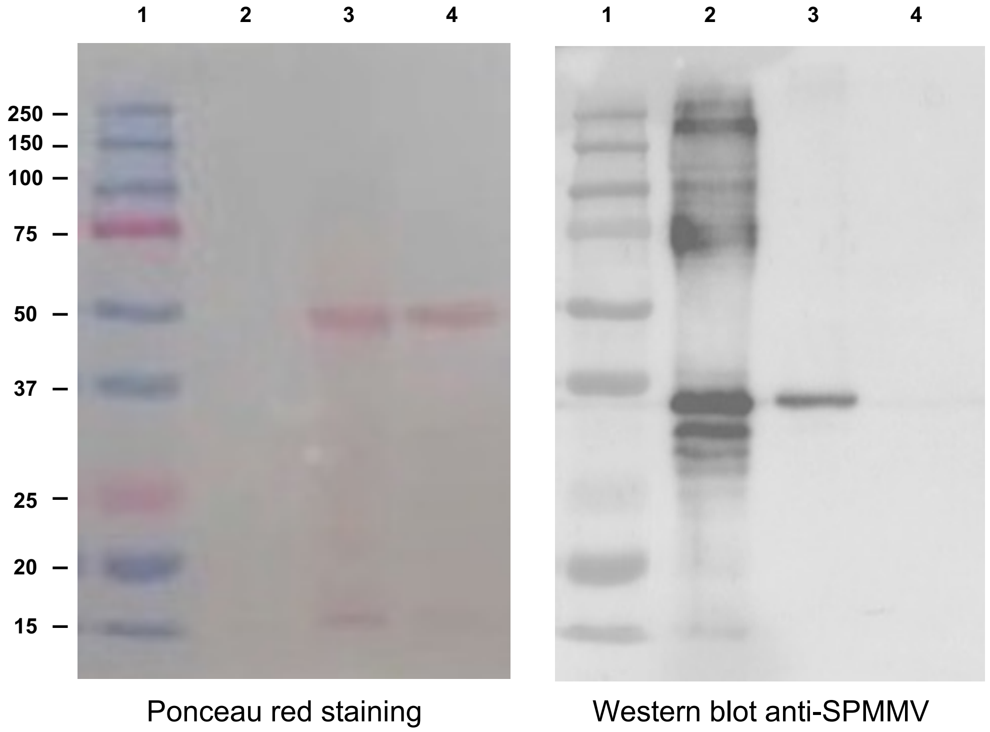


Ponceau red staining (left) and Western blot analysis (right) of samples corresponding to purified SPMMV-VLPs (lanes 2), and total protein extracts of tobacco plants infected with SPMMV (lanes 3) or non-inoculated control (lanes 4). The prestained molecular weigth markers (Precision Plus Protein standard, BioRad) are shown with their sizes in kDa indicated (lanes 1). The Western blot was incubated with Polyclonal IgG antibody against SPMM-AS0900 (DMSZ) and revealed with the corresponding antirabbit conjugate. The 34 kDa band in the VLPs sample shows identical mobility as the CP in the infected sample, with the lower bands around 30 kDa being also recognized by the antibody.

**Supplementary Fig. 4: SPFMV and SPV2 VLPs encapsidate pEff-derived RNA.**

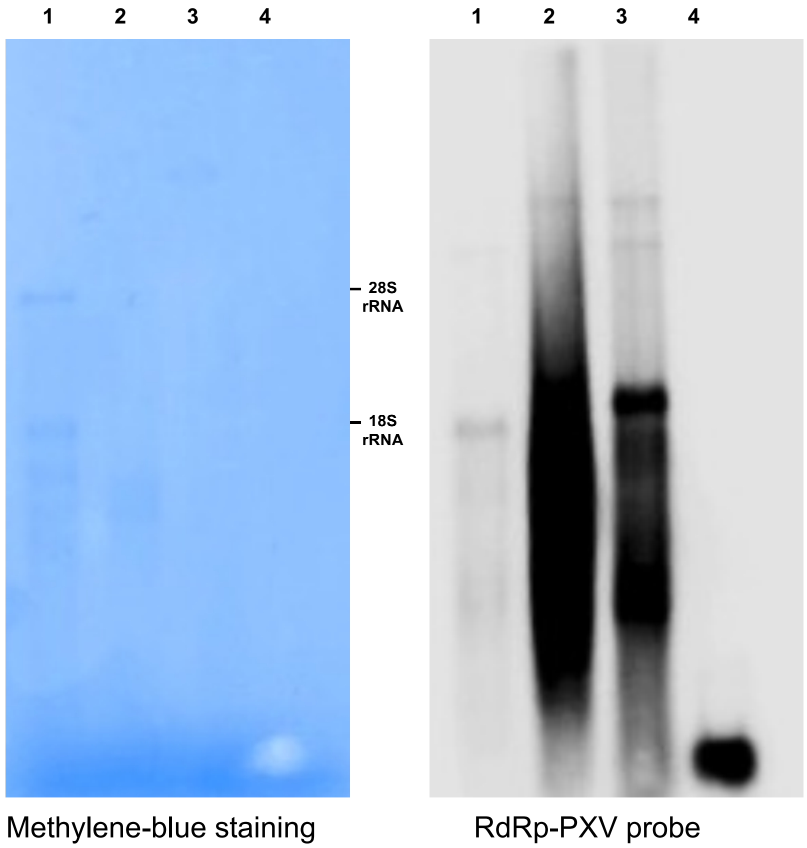


Methylene-blue staining (left) and northern blot analysis (right) of samples corresponding to 1µg of RNA extracted from infiltrated tissue with pEff vector (lanes 1), purified SPFMV-VLPs (lanes 2) and purified SPMMV-VLPs (lanes 3), using a probe corresponding to a region of the RdRp of PVX included in pEff sequence, revealing the presence of vector-derived RNA in both types of VLPs. A PCR product (DNA) of PVX RdRp encompassing the probe binding site was included as positive control for the hybridization (lane 4), and the mobilities of 28S and 18S rRNAs, corresponding respectively to 4,000 and 1,600 nts, are indicated as internal size markers. The size of PCR product was 222 bp and it was obtained with primers PVX-probeF1 and PVX-probeR1 as previously described (Thuenemann et al., 2021).

**Supplementary Fig. 5: Comparison of *Potyviridae* and *Alphaflexviridae* coat protein structures.**

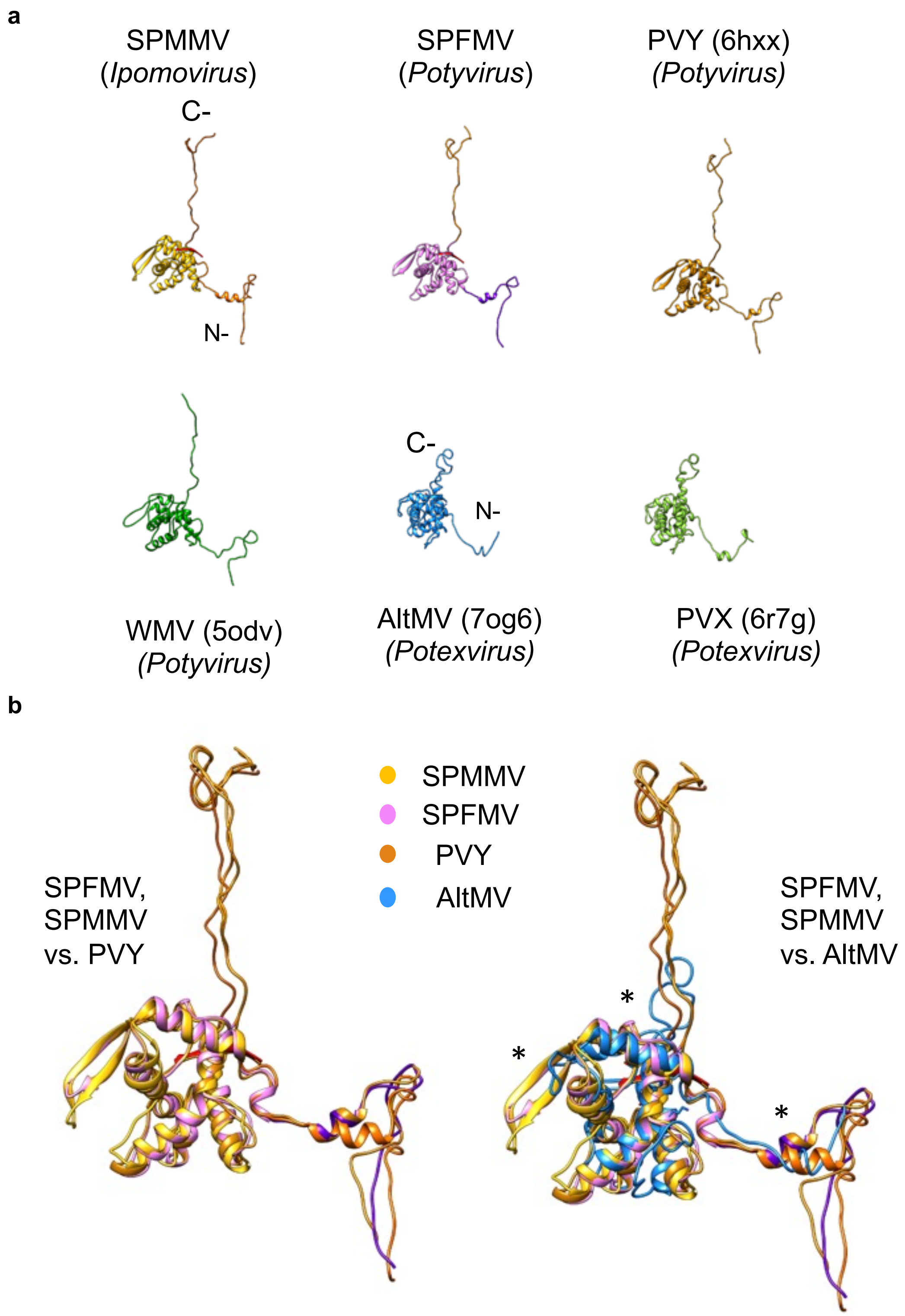


**a** Reported coat protein structures of selected helical viruses (indicated) within the *Potyviridae* and *Alphaflexiviridae* families. **b** Aligned structures of the coat proteins of three *Potyviridae* members (SPMMV, SPFMV and PVY) and the *Alphaflexviridae* family member AltMV, identified using the color code in the middle. Key structural differences between the two families are indicated by an asterisk.

**Supplementary Fig. 6: Alphafold structure predictions.**

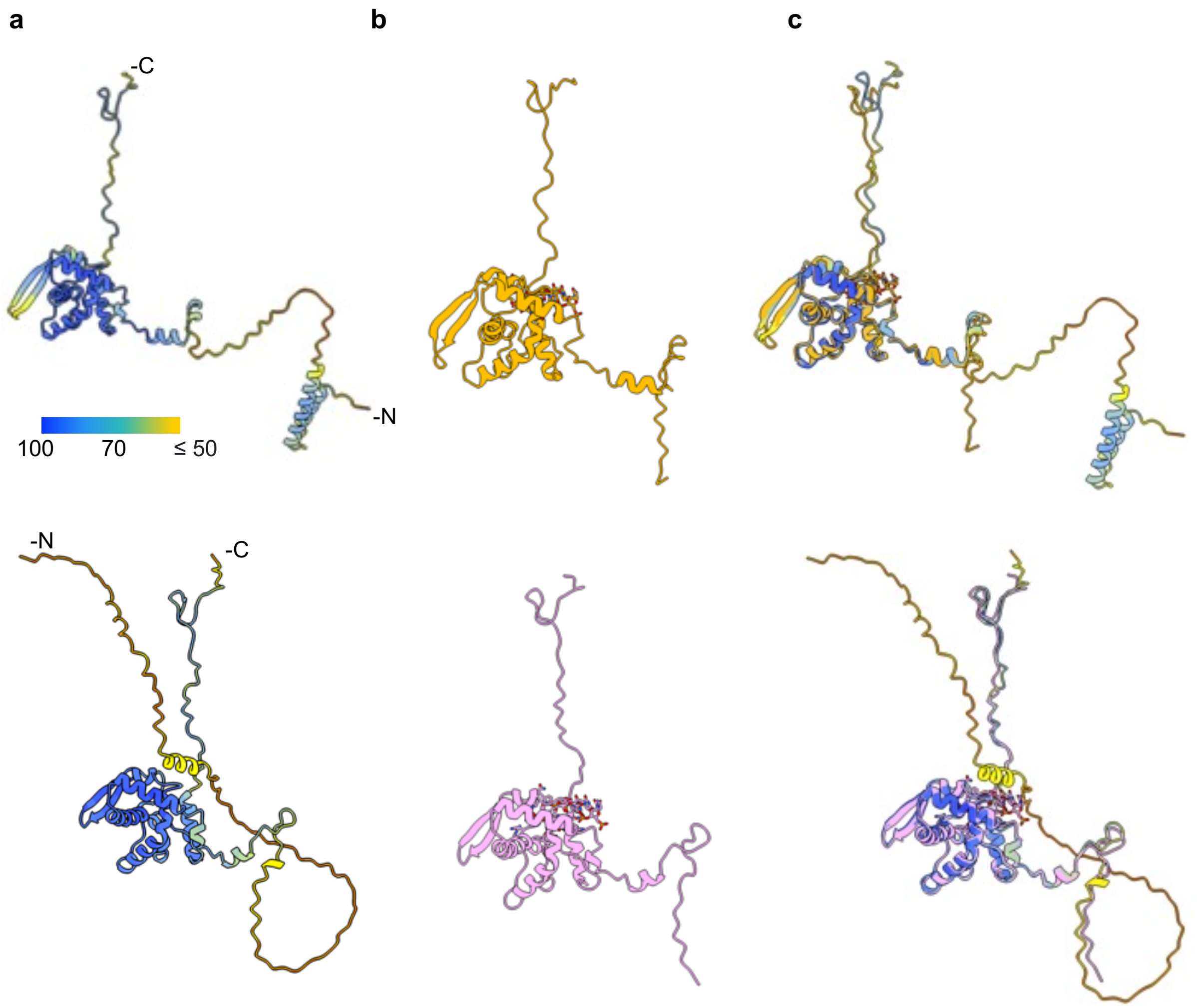


**a** Alphafold predicted CP structures for SPFMV (top panel), and for SPMMV (bottom panel). The models are coloured according to the pLDDT scores, with colour bar indicated (scores of 100-70 coloured blue to green indicate high confidence, and 50-0 coloured yellow to orange indicate low confidence). **b** CryoEM structures for CP of SPFMV (top panel, in gold) and of SPMMV (bottom panel, in pink). **c** Aligned structures from cryoEM and alphafold prediction analysis of SPFMV (top panel) and SPMMV (bottom panel).

**Supplementary Fig. 7: CryoEM image processing workflow.**

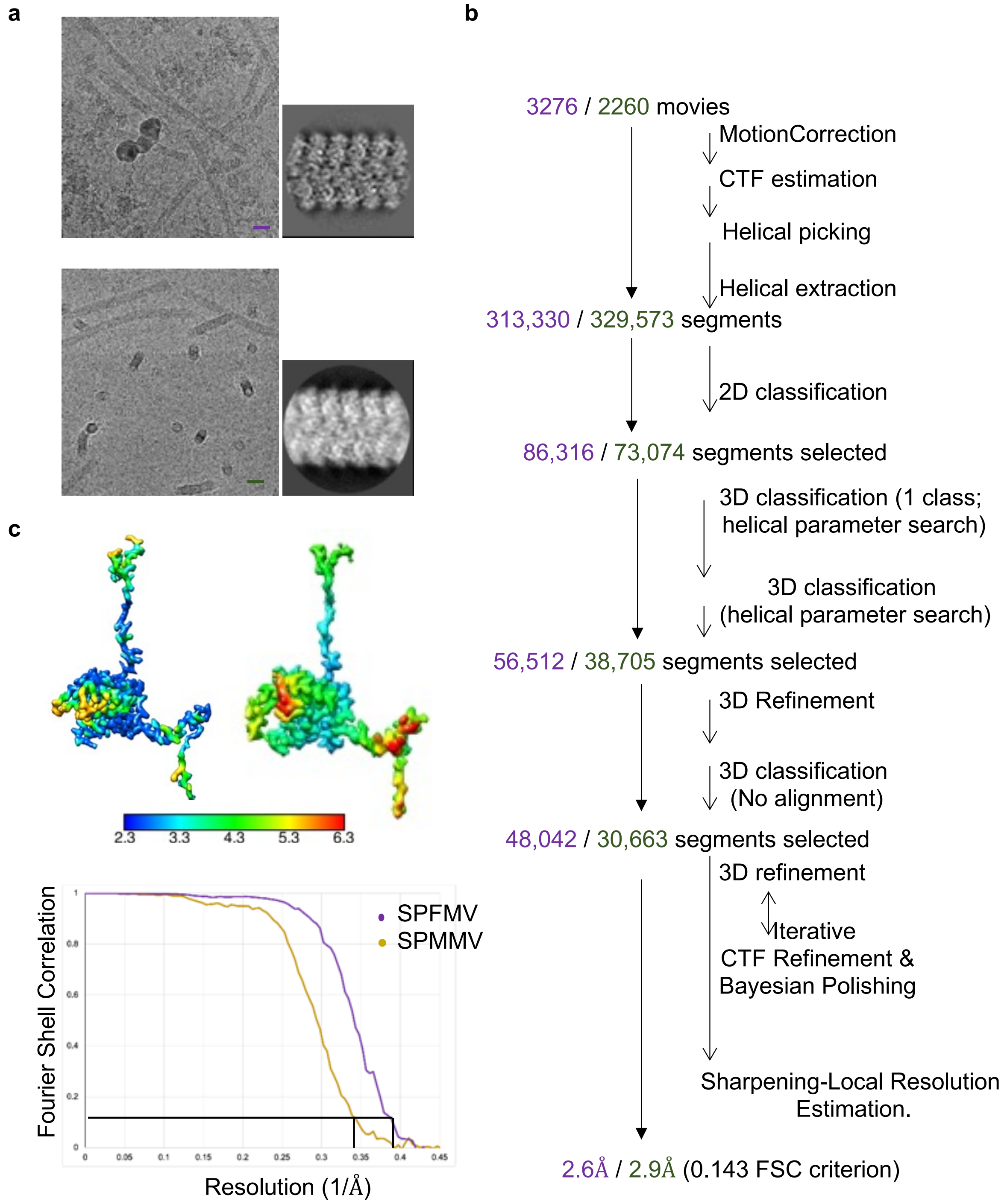


**a** Representative cryoEM micrograph and 2D class average of the helical segment from SPFMV (top panel) and SPMMV (bottom panel). Scale bars correspond to 20 nm. **b** Workflow for image processing. **c** Local resolution maps for the CPs of SPFMV (left) and SPMMV (right), with the graph showing the masked fourier-shell correlation (FSC) curves for the two structures.

**Supplementary Fig. 8: Uncropped versions of gels and blots in the Figures 1 and 2, and in Supplementary Figures 1, 3 and 4**

Figure 1, panel b, Western blot

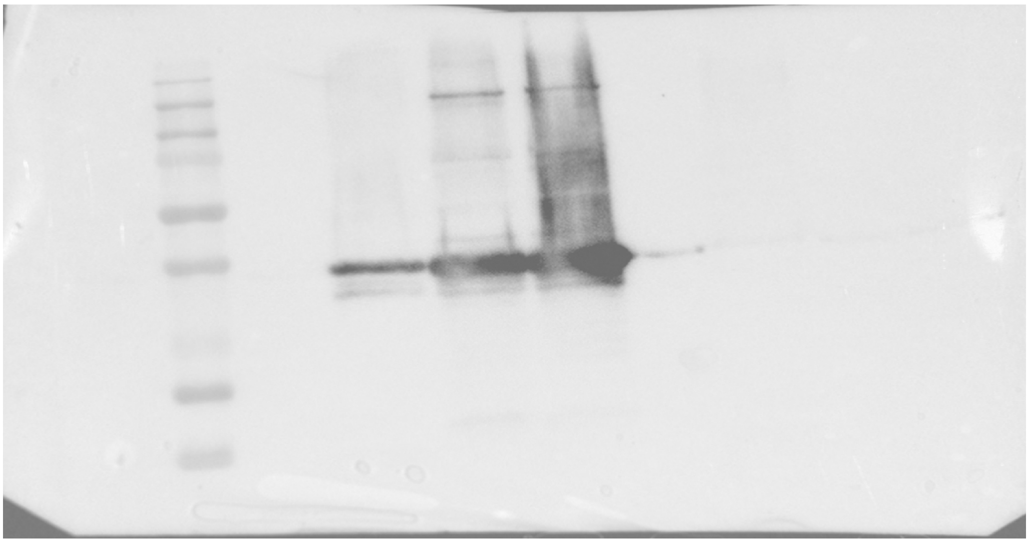

Figure 1, panel b, Ponceau staining (loading control)

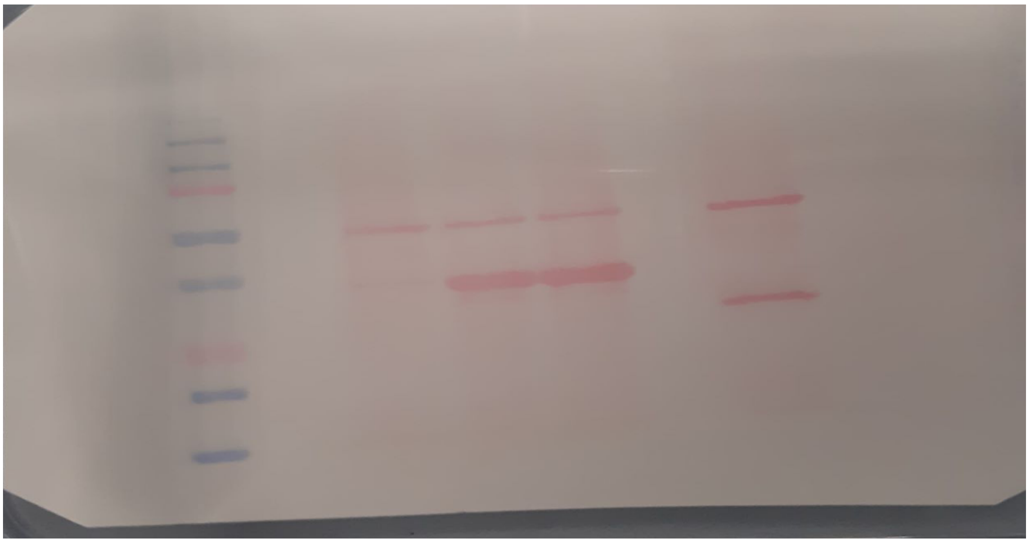

Figure 1, panel c, Western blot

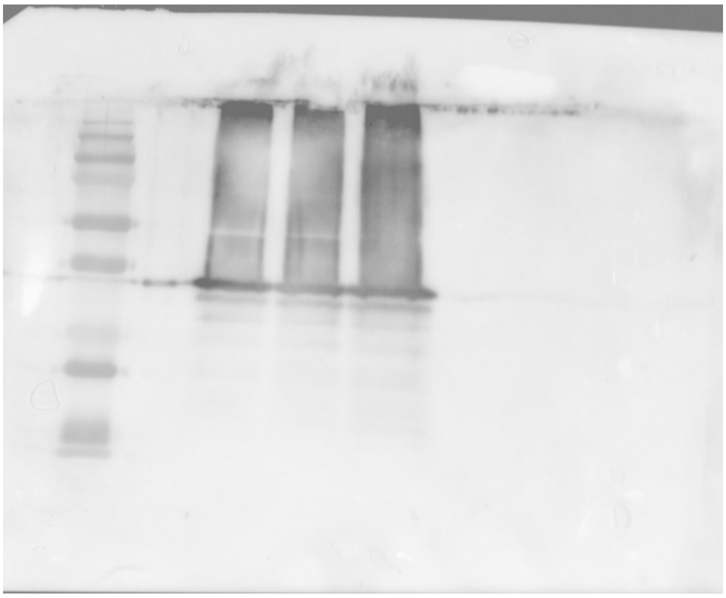

Figure 1, panel c, Ponceau staining (loading control)

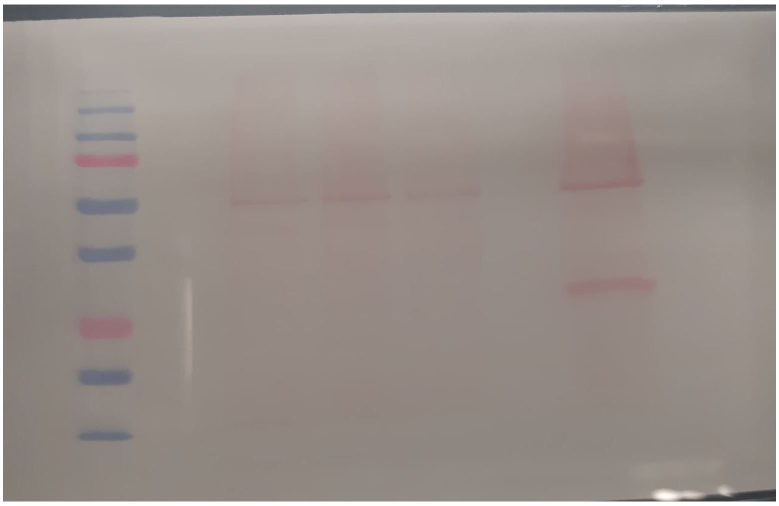

Figure 2, panel a, Coomassie staining SDS-PAGE gel

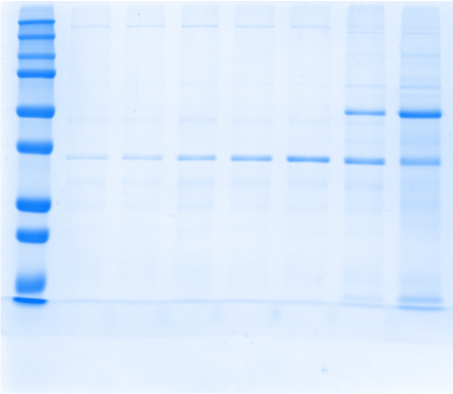

Figure 2, panel c, Coomassie staining SDS-PAGE gel

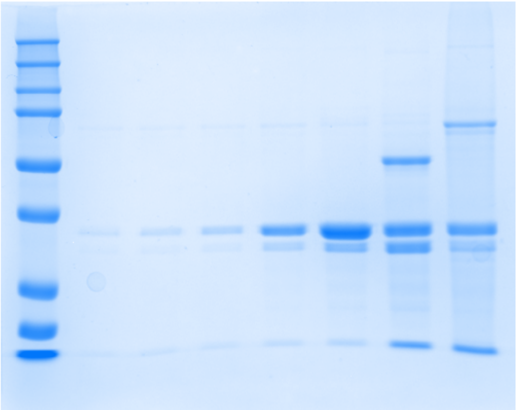

Figure 2, panel e, Methylene blue staining (left) and Northern blot (right)

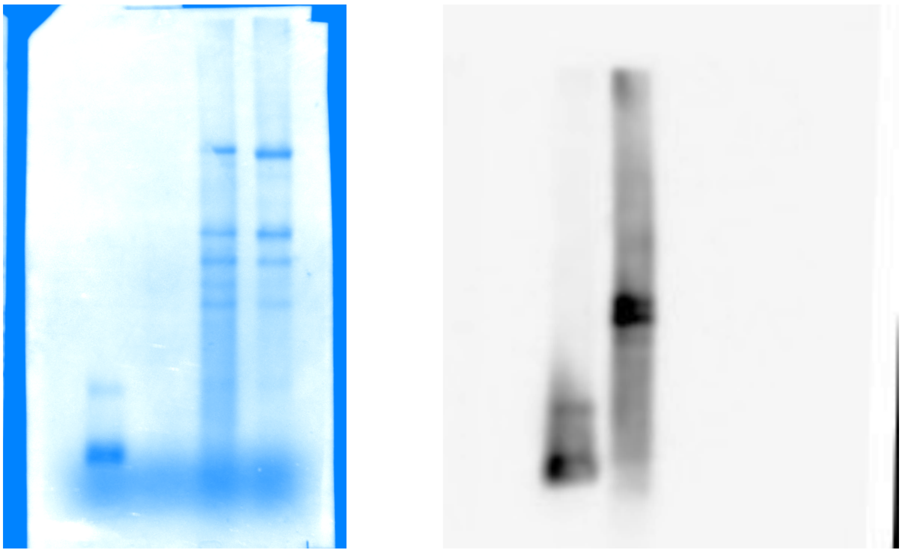

Figure 2, panel f, Methylene blue staining (left) and Northern blot (right)

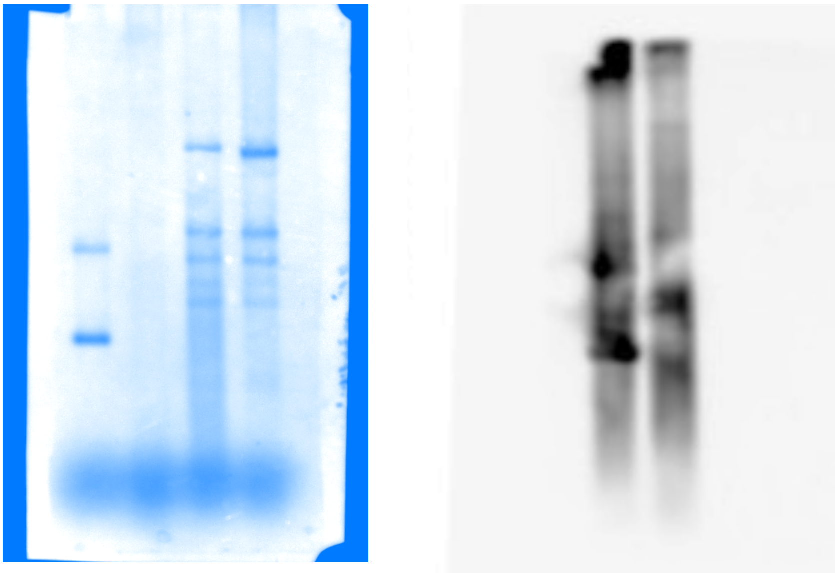

Supplementary Fig. 1, panel a, Western blot

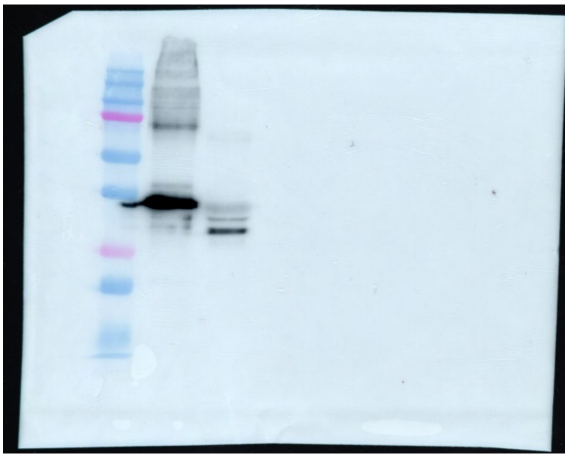

Supplementary Fig. 1, panel b, Western blot

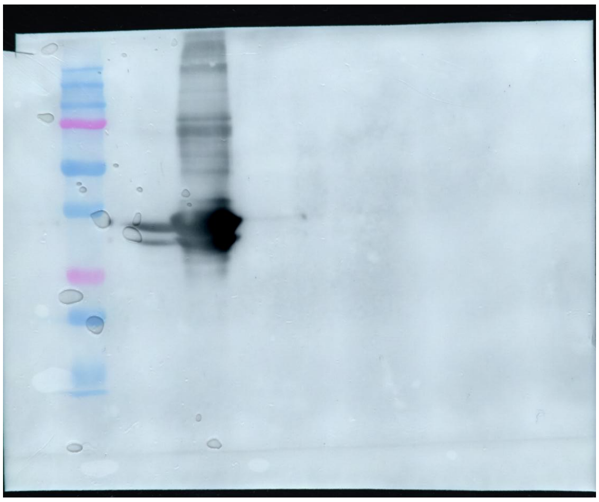

Supplementary Fig. 3, Ponceau staining

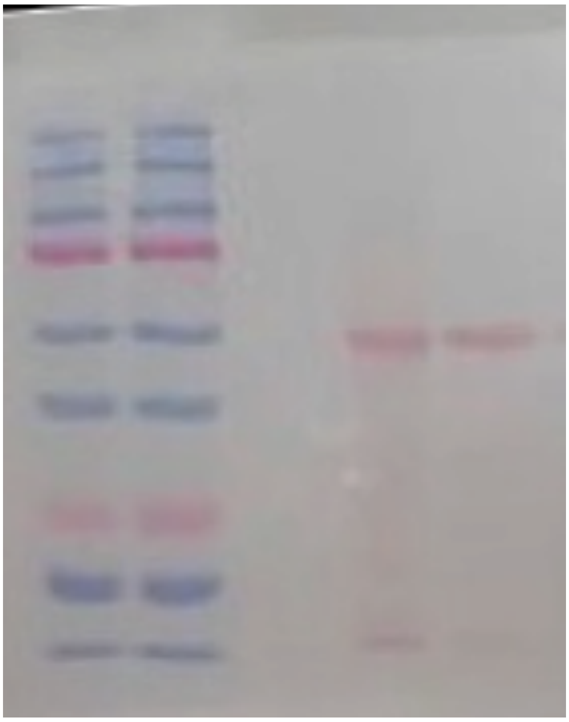

Supplementary Fig. 3, Western blot

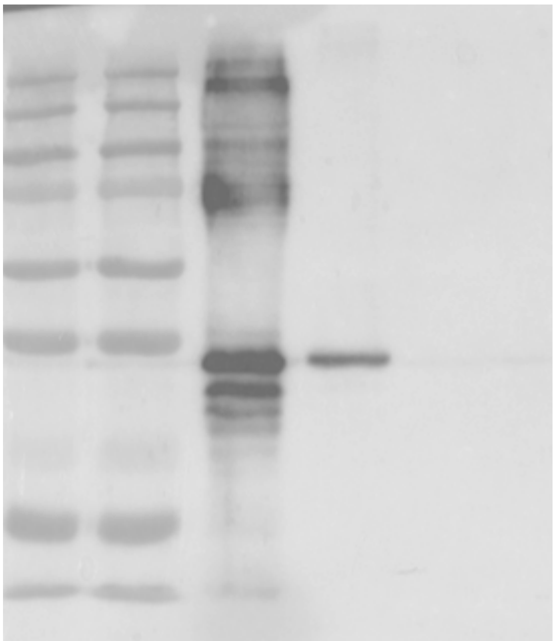

Supplementary Fig. 4, Methylene blue staining (left) and Northern blot (right)

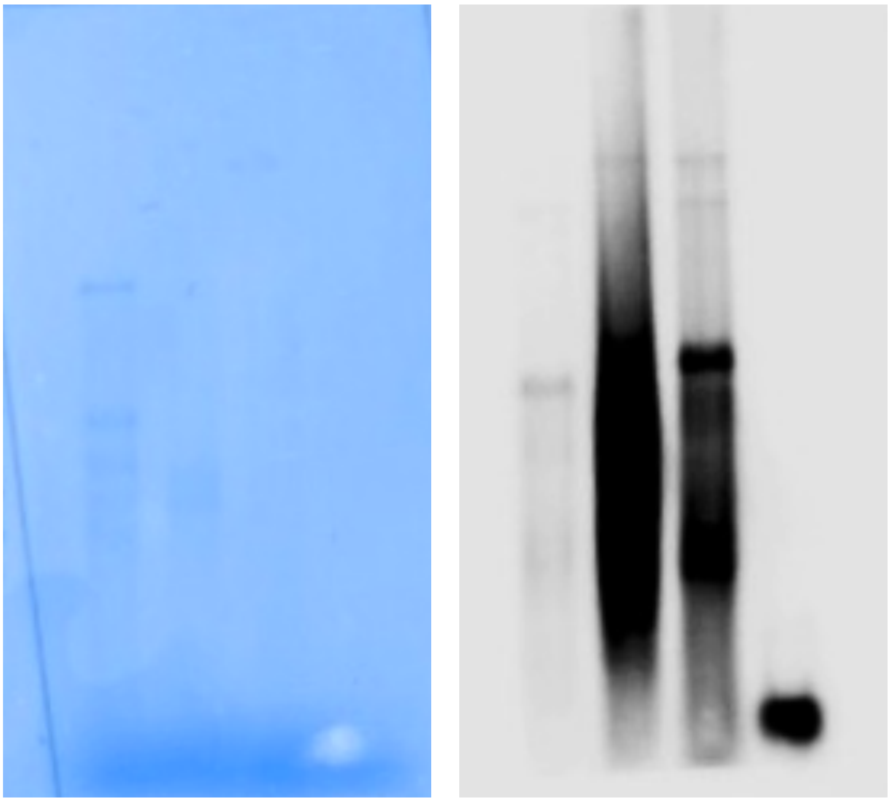

**Supplementary Table 1. Reports of SPFMV CP subunit interactions to subunits adjacent (N and N+1) and in the next layer (N and N+10) of the helical structure.**

SPFMV_N & N+1

| | Hydrogen Bonds | | | | |
|---|---|---|---|---|---|
| Interaction | CP (N-1) | Atom | Distance Å | CP (N) | Atom |
| 1 | Gln 103 | N | 3.1 | Asp 212 | OD2 |
| 2 | Asn 113 | N | 3.81 | Gly 156 | O |
| 3 | Gln 124 | NE2 | 2.51 | Met 228 | O |
| 4 | Gln 124 | NE2 | 2.85 | Glu 168 | OE1 |
| 5 | Asn 129 | N | 3.38 | Asn 169 | OD1 |
| 6 | Ser 254 | OG | 2.59 | Ala 226 | O |
| 7 | Asn 304 | N | 3.22 | Asp 300 | OD1 |
| 8 | Leu 308 | N | 3.72 | Ala 305 | O |
| 9 | Ala 309 | N | 3.26 | Leu 308 | O |
| 10 | Pro 104 | O | 3 | Gln 134 | NE2 |
| 11 | Asn 113 | O | 3.56 | Asn 160 | ND2 |
| 12 | Tyr 120 | O | 2.83 | Tyr 217 | OH |
| 13 | Tyr 120 | OH | 3.25 | Met 182 | N |
| 14 | Pro 122 | O | 2.84 | Arg 221 | NH2 |
| 15 | Glu 126 | O | 3.42 | Thr 180 | OH1 |
| 16 | Val 127 | O | 3.13 | Asn 169 | ND2 |
| 17 | Asn 129 | OD1 | 2.74 | Arg 230 | NH2 |
| 18 | Ala 307 | O | 3.84 | Ala 307 | N |
| 19 | Ala 309 | O | 3.42 | Gly 310 | N |

| | | Salt bridge | | | |
|---|---|---|---|---|---|
| Interaction | CP (N-1) | Atom | Distance Å | CP (N) | Atom |
| 1 | Arg 109 | NH2 | 3.84 | Asp 152 | OD1 |
| 2 | Arg 109 | NH3 | 3.91 | Asp 152 | OD2 |

SPFMV_N & N+10

| | Hydrogen Bonds | | | | |
|---|---|---|---|---|---|
| Interaction | CP (N-1) | Atom | Distance Å | CP (N) | Atom |
| 1 | Arg 93 | NH1 | 3.38 | Met 182 | O |
| 2 | Lys 95 | NZ | 3.22 | Glu 186 | OE2 |
| 3 | Lys 95 | NZ | 2.97 | Asp 183 | OD2 |
| 4 | Asn 97 | N | 3.49 | Tyr 148 | O |
| 5 | Val 94 | O | 3.09 | Tyr 190 | OH |

| | | Salt Bridges | | | |
|---|---|---|---|---|---|
| Interaction | CP (N-1) | Atom | Distance Å | CP (N) | Atom |
| 1 | Arg 93 | NE | 3.94 | Asp 183 | OD1 |
| 2 | Arg 93 | NH2 | 3.64 | Asp 183 | OD1 |
| 3 | Lys 95 | NZ | 3.22 | Glu 186 | OE2 |
| 4 | Lys 95 | NZ | 2.97 | Asp 183 | OD2 |

**Supplementary Table 2. Reports of SPMMV CP subunit interactions to subunits adjacent (N and N+1) and in the next layer (N and N+10) of the helical structure.**

## SPMMV_N & N+1

| | Hydrogen Bonds | | | | |
|---|---|---|---|---|---|
| Interaction | CP (N-1) | Atom | Distance Å | CP (N) | Atom |
| 1 | Ile 80 | N | 2.89 | Glu 199 | OE2 |
| 2 | Arg 82 | NH1 | 2.05 | Glu 113 | OE1 |
| 3 | Gln 83 | NE2 | 3.03 | Glu 113 | O |
| 4 | Gln 84 | NE2 | 3.62 | Gln 200 | OE1 |
| 5 | Glu 113 | N | 3.34 | Tyr 176 | OH |
| 6 | Ala 114 | N | 2.64 | Tyr 176 | OH |
| 7 | Gln 84 | OE1 | 2.71 | Gln 20 | NE2 |
| 8 | Ile 96 | O | 3.87 | Gln 204 | NE2 |
| 9 | Tyr 99 | OH | 3.58 | Val 164 | N |
| 10 | Ser 103 | O | 3.66 | Thr 216 | OG1 |
| 11 | Ser 103 | OG | 2.34 | Thr 216 | N |
| 12 | Glu 107 | O | 3 | Arg 162 | NH2 |
| 13 | Leu 110 | O | 3.13 | Arg 162 | NH2 |
| 14 | Thr 241 | O | 3.41 | Thr 215 | OG1 |

| | | Salt bridges | | | |
|---|---|---|---|---|---|
| Interaction | CP (N-1) | Atom | Distance Å | CP (N) | Atom |
| 1 | Arg 82 | NH1 | 2.05 | Glu 113 | OE1 |

## SPMMV_N & N+10

| | Hydrogen Bonds | | | | |
|---|---|---|---|---|---|
| Interaction | CP (N-1) | Atom | Distance Å | CP (N) | Atom |
| 1 | Ala66 | O | 3.14 | Met 166 | N |

**Supplementary Table 3. Reports for SPFMV and SPMMV CP subunit interactions to the RNA.**

## SPFMV_N & RNA

| | Hydrogen bonds | | | | |
|---|---|---|---|---|---|
| Interaction | CP (N-1) | Atom | Distance Å | CP (N) | Atom |
| 1 | U5 | N3 | 3.42 | Asn 174 | O |
| 2 | U1 | O3' | 3.41 | Gln 205 | NE2 |
| 3 | U2 | OP1 | 2.73 | Gln 205 | NE2 |
| 4 | U3 | OP3 | 2.79 | Arg 204 | NH1 |
| 5 | U4 | OP1 | 3.02 | Arg 204 | NH1 |
| 6 | U4 | OP1 | 3.18 | Arg 204 | NH2 |
| 7 | U4 | OP2 | 2.6 | Ser 172 | OH |
| 8 | U4 | O3' | 3.89 | Arg 235 | NH1 |
| 9 | U4 | O2 | 2.23 | Tyr 231 | OH |
| 10 | U5 | OP1 | 3.18 | Arg 235 | NH1 |
| 11 | U5 | OP1 | 3.05 | Arg 235 | NH2 |
| 12 | U5 | O3' | 2.89 | Gly 170 | N |

## SPMMV_N & RNA

| | Hydrogen bonds | | | | |
|---|---|---|---|---|---|
| Interaction | CP (N-1) | Atom | Distance Å | CP (N) | Atom |
| 1 | U2 | OP2 | 2.9 | Arg 239 | NH2 |
| 2 | U2 | O3' | 3.04 | Gln 192 | NE2 |
| 3 | U2 | O2' | 3.87 | Gln 192 | NE2 |
| 4 | U3 | OP2 | 2.91 | Arg 195 | NH2 |
| 5 | U3 | OP2 | 2.87 | Gln 192 | NE2 |
| 6 | U3 | O3' | 3.71 | Gln 153 | NE2 |
| 7 | U3 | O2' | 2.72 | Lys 264 | NZ |
| 8 | U4 | O2' | 3.84 | Lys 264 | NZ |
| 9 | U5 | OP1 | 3 | Ser 152 | OG |

**Note:** The reports were generated by PISA (Proteins, Interfaces, Structures and Assemblies) analysis of structures with interactions based on a pre-established cutoff for distance-based hydrogen bonds, showing interactions only when the distance between donor and acceptor atoms is less than 3.89A. Therefore, residues Asp248 (SPFMV), and Arg262 and Asp 236 (SPMMV) which are present within the corresponding binding pockets as shown in figure 6, appear to be at a calculated distance away of the RNA.

## Supplementary Table 4. List of primers

| Primer name | Sequence (5´- 3´) | Amplicon size |
|---|---|---|
| pEff-CP-SPFMV-FW | TACTTCCATCAGGCGCGCCATGTCTAGTGAGAGCACTGA | 988 bp |
| pEff-CP-SPFMV-RV | ATTACTTGTACACCCGGGTCATTGCACACCCCTCATTC | |
| pEff-CP-SPMMV-FW | ACTTCCATCAGGCGCGCCATGTCGACATCCAAGACAAT | 946 bp |
| pEff-CP-SPMMV-RV | GATTACTTGTACACCCGGGTCAGTCGAGTTGAGCTCCTC | |
| PVX-probeF1 | CTCACAGAGATCACAAC GGAAAC | 222 bp |
| PVX-probeR1 | taatacgactcactatagggATGGT TGCCTGGTATATACTGGA AAC | |

180
