# Supplementary material for: CryoEM and stability analysis of virus-like particles of potyvirus and ipomovirus infecting a common host
Source: Commun Biol. 2023 Apr 19;6:433. doi: 10.1038/s42003-023-04799-x (PMC10115852; doi:10.1038/s42003-023-04799-x)
Supplement: Supplementary file 3 — Description of Additional Supplementary Files [file 42003_2023_4799_MOESM3_ESM.pdf]

Description of Additional Supplementary Files

File name: Supplementary Data 1

Description: Source Data for the thermal stability assay of purified VLPs of SPFMV and SPMMV (depicted in Figure 3).
