# Supplementary material for: CryoEM and stability analysis of virus-like particles of potyvirus and ipomovirus infecting a common host
Source: Commun Biol. 2023 Apr 19;6:433. doi: 10.1038/s42003-023-04799-x (PMC10115852; doi:10.1038/s42003-023-04799-x)
Supplement: Supplementary file 5 — Reporting Summary [file 42003_2023_4799_MOESM5_ESM.pdf]

nature portfolio

Corresponding author(s): Juan Jose Lopez-Moya

Last updated by author(s): Apr 3, 2023

Reporting Summary

Nature Portfolio wishes to improve the reproducibility of the work that we publish. This form provides structure for consistency and transparency in reporting. For further information on Nature Portfolio policies, see our Editorial Policies and the Editorial Policy Checklist.

Statistics

For all statistical analyses, confirm that the following items are present in the figure legend, table legend, main text, or Methods section.

n/a	Confirmed	
☐	☒	The exact sample size (*n*) for each experimental group/condition, given as a discrete number and unit of measurement
☐	☒	A statement on whether measurements were taken from distinct samples or whether the same sample was measured repeatedly
☒	☐	The statistical test(s) used AND whether they are one- or two-sided<br>*Only common tests should be described solely by name; describe more complex techniques in the Methods section.*
☒	☐	A description of all covariates tested
☒	☐	A description of any assumptions or corrections, such as tests of normality and adjustment for multiple comparisons
☒	☐	A full description of the statistical parameters including central tendency (e.g. means) or other basic estimates (e.g. regression coefficient) AND variation (e.g. standard deviation) or associated estimates of uncertainty (e.g. confidence intervals)
☒	☐	For null hypothesis testing, the test statistic (e.g. *F*, *t*, *r*) with confidence intervals, effect sizes, degrees of freedom and *P* value noted<br>*Give P values as exact values whenever suitable.*
☐	☒	For Bayesian analysis, information on the choice of priors and Markov chain Monte Carlo settings
☒	☐	For hierarchical and complex designs, identification of the appropriate level for tests and full reporting of outcomes
☒	☐	Estimates of effect sizes (e.g. Cohen's *d*, Pearson's *r*), indicating how they were calculated

Our web collection on statistics for biologists contains articles on many of the points above.

Software and code

Policy information about availability of computer code

Data collection	ThermoFischer Scientific EPU software was used for automated cryoEM data collection.
Data analysis	RELION 3.1, MotionCorr2, CTFFIND4, crYOLO were used for image processing.<br>Post processing: We used RELION 3.1, ResMap (for local resolution estimation),<br>Modelling: Phyre 2 and Alphafold were used for homology modelling and sequence-based structure predictions respectively.<br>UCSF Chimera, ISOLDE, Phenix and Coot were used for atomic model building, fitting and structure refinement.

For manuscripts utilizing custom algorithms or software that are central to the research but not yet described in published literature, software must be made available to editors and reviewers. We strongly encourage code deposition in a community repository (e.g. GitHub). See the Nature Portfolio guidelines for submitting code & software for further information.

Data

Policy information about availability of data

All manuscripts must include a data availability statement. This statement should provide the following information, where applicable:

- Accession codes, unique identifiers, or web links for publicly available datasets
- A description of any restrictions on data availability
- For clinical datasets or third party data, please ensure that the statement adheres to our policy

The cryoEM maps were deposited to the electron microscopy databank (EMDB) with accession codes 8ACB (SPFMV) and 8ACC (SPMMV), and the models deposited

to protein data bank (PDB) with accession codes of EMD-15345 (SPFMV) and EMD-15346 (SPMMV) respectively. The raw data from which these models were derived are deposited in the EMPIAR database as EMPIAR-11490 - SPMMV and EMPIAR-11491 - SPFMV.

Human research participants

Policy information about studies involving human research participants and Sex and Gender in Research.

Reporting on sex and gender	Work refers to plant virus structures, not requiring to consider any sex or gender aspects
Population characteristics	Work refers to plant virus structures, not requiring to consider a population
Recruitment	Work refers to plant virus structures, not requiring to recruit individuals
Ethics oversight	Work refers to plant virus structures, not requiring ethics oversight

Note that full information on the approval of the study protocol must also be provided in the manuscript.

Field-specific reporting

Please select the one below that is the best fit for your research. If you are not sure, read the appropriate sections before making your selection.

☒ Life sciences ☐ Behavioural & social sciences ☐ Ecological, evolutionary & environmental sciences

For a reference copy of the document with all sections, see nature.com/documents/nr-reporting-summary-flat.pdf

Life sciences study design

All studies must disclose on these points even when the disclosure is negative.

Sample size	Description of virus-like particles lengths was determined with a sample of 150 individual in well contrasted TEM images
Data exclusions	No exclusions were considered
Replication	Experiments were reproduced when considered necessary
Randomization	Not relevant for determination of structures
Blinding	Not relevant for determination of structures

Reporting for specific materials, systems and methods

We require information from authors about some types of materials, experimental systems and methods used in many studies. Here, indicate whether each material, system or method listed is relevant to your study. If you are not sure if a list item applies to your research, read the appropriate section before selecting a response.

Materials & experimental systems

n/a	Involved in the study	
☐	☒	Antibodies
☒	☐	Eukaryotic cell lines
☒	☐	Palaeontology and archaeology
☒	☐	Animals and other organisms
☒	☐	Clinical data
☒	☐	Dual use research of concern

Methods

n/a	Involved in the study	
☒	☐	ChIP-seq
☒	☐	Flow cytometry
☒	☐	MRI-based neuroimaging

Antibodies

Antibodies used	Polyclonal antibodies anti-SPFMV-CP (RT-0898 DMSZ, Braunschweig, Germany), anti-SPMMV-CP (RT-0900 DMSZ, Braunschweig, Germany) and Goat anti-rabbit horseradish peroxide (HRP) secondary antibody (Cat. 31460, InvitrogenTM, Thermo Fischer Scientific)
Validation	Western blot analysis
